# Supplementary material for: Assessment of locomotive syndrome among older individuals: a confirmatory factor analysis of the 25-question Geriatric Locomotive Function Scale
Source: PeerJ. 2020 Apr 14;8:e9026. doi: 10.7717/peerj.9026 (PMC7164427; doi:10.7717/peerj.9026)
Supplement: Supplemental Information 1 [file peerj-08-9026-s001.docx]

| **Supplementary Table. 1: Descriptive statistics for the 25-question Geriatric Locomotive Function Scale (GLFS-25), n of participants = 500.** | | | | | | |
| --- | --- | --- | --- | --- | --- | --- |
|  | **Mean** | **SD** | **Min** | **Max** | **Skew** | **Kurtosis** |
| **Age** | 72.64 | 7.41 | 60 | 91 | 0.26 | -1.01 |
| **Q1** | 1.39 | 1.14 | 0 | 4 | 0.42 | -0.76 |
| **Q2** | 1.33 | 1.13 | 0 | 4 | 0.47 | -0.77 |
| **Q3** | 1.36 | 1.16 | 0 | 4 | 0.56 | -0.55 |
| **Q4** | 1.21 | 1.07 | 0 | 4 | 0.51 | -0.76 |
| **Q5** | 0.67 | 0.92 | 0 | 4 | 1.40 | 1.41 |
| **Q6** | 0.59 | 0.85 | 0 | 4 | 1.33 | 0.96 |
| **Q7** | 0.50 | 0.80 | 0 | 4 | 1.55 | 1.71 |
| **Q8** | 0.35 | 0.68 | 0 | 3 | 1.95 | 3.11 |
| **Q9** | 0.52 | 0.77 | 0 | 4 | 1.59 | 2.58 |
| **Q10** | 0.35 | 0.70 | 0 | 4 | 2.23 | 5.25 |
| **Q11** | 0.45 | 0.78 | 0 | 4 | 1.86 | 3.34 |
| **Q12** | 1.12 | 1.13 | 0 | 4 | 0.82 | -0.23 |
| **Q13** | 1.36 | 1.25 | 0 | 4 | 0.64 | -0.65 |
| **Q14** | 0.44 | 0.79 | 0 | 4 | 1.82 | 2.62 |
| **Q15** | 1.18 | 1.20 | 0 | 4 | 0.85 | -0.19 |
| **Q16** | 0.56 | 0.89 | 0 | 4 | 1.76 | 2.90 |
| **Q17** | 0.93 | 1.16 | 0 | 4 | 1.14 | 0.28 |
| **Q18** | 0.84 | 1.14 | 0 | 4 | 1.28 | 0.66 |
| **Q19** | 0.61 | 0.89 | 0 | 4 | 1.48 | 1.59 |
| **Q20** | 1.05 | 1.16 | 0 | 4 | 0.95 | -0.09 |
| **Q21** | 1.45 | 1.36 | 0 | 4 | 0.66 | -0.84 |
| **Q22** | 0.78 | 1.12 | 0 | 4 | 1.43 | 1.08 |
| **Q23** | 1.35 | 1.40 | 0 | 4 | 0.78 | -0.72 |
| **Q24** | 0.74 | 0.98 | 0 | 4 | 1.60 | 2.35 |
| **Q25** | 1.16 | 1.13 | 0 | 4 | 0.94 | 0.05 |
| Abbreviations: SD, standard deviation; Min, minimum; Max, maximum. | | | | | | |
